# Keeping up appearances: Don’t frown upon the effects of botulinum toxin injections in facial muscles

**DOI:** 10.1016/j.cnp.2023.05.005

**Published:** 2023-08-09

**Authors:** Anna Rostedt Punga, Mohammad Alimohammadi, Maarika Liik

**Affiliations:** a Department of Medical Sciences, Clinical Neurophysiology, Uppsala University and Uppsala University Hospital, Uppsala, Sweden; b Department of Medical Sciences, Dermatology and Venerology, Uppsala University and Uppsala University Hospital, Uppsala, Sweden

**Keywords:** Botulinum toxin, Neuromuscular, Long-term effects, Cosmetic

## Abstract

Aesthetic use of low doses of Botulinum toxin (BoNT) injections into the facial muscles has become a leading non-surgical aesthetic treatment worldwide to reduce facial wrinkles, including glabellar lines, forehead lines, and periorbital wrinkles. Within these aesthetic applications, BoNT injections intend to reduce and prevent wrinkles, and the recommended usage of 2 years is often exceeded, which may result in atrophy of the injected muscles. The long-term effects of BoNT injections in the facial muscles and the evidence of diffusion of BoNT to surrounding muscles are obvious pitfalls and challenges for clinical neurophysiologists in differential diagnosing neuromuscular transmission failures. Also, this is further complicated by the risk of developing side effects upon permanent chemical denervation of facial muscles, with less possibility for reinnervation.

This review summarizes the known long-term effects of BoNT over time in different facial muscles and the use of objective electrophysiological measures to evaluate these. A better understanding of the long-term effects of BoNT is essential to avoid misdiagnosing other neuromuscular disorders.

## Highlights

• Aesthetic botulinum toxin injections may result in atrophy of the injected facial muscles.
• The long-term effects and diffusion of botulinum toxin in the facial muscles are pitfalls in the neurophysiological diagnosis.
• Serial injections of botulinum toxin in the facial muscles may cause permanent chemical denervation.

## 1 Introduction

Botulinum toxin type A (BoNTA) causes neuromuscular transmission failure by blocking the pre-synaptic fusion of acetylcholine-containing vesicles and thereby inhibiting acetylcholine release from the motor nerve into the neuromuscular junction (Blasi et al., 1993). BoNTA is the most potent serotype of botulinum toxins, possessing a toxicity of one million-fold higher than cobra toxin and far higher than cyanide (Willis et al., 2008). The number of research articles on PubMed on botulinum toxin has exploded in recent years, particularly the different new treatment areas for BoNTA in the cosmetic and medical fields. Treatment with botulinum-toxin is one cosmetic procedure that provides the highest value for money in terms of aesthetic improvement. The desired cosmetic effect is archived by paralysis and atrophy of delicate facial muscles. In addition, serial and repeated injections may also cause undesirable and unanticipated muscle atrophy that is impossible to reverse. Since the pharmacological indications for BoNTA use are short-term, there needs to be documented long-term effects in this area. While BoNTA injections are generally considered safe, their extensive and growing use in cosmetic applications raises concerns about safety and adverse events mimicking neuromuscular disorders (Punga and Liik, 2020).

This article reviews the characteristic findings after cosmetic injections of BoNTA in facial muscles, both short-term and long-term.

## 2 Methodology: Search strategy and selection criteria

Using a comprehensive search syntax (MeSH and free-text terms), we searched PubMed using the following terms: “botulinum toxin” or “BoNTA” and “cosmetic” or “neurophysiological”. The search was performed on Dec 13, 2022, with no strict start date. Still, papers of all study design with a publication date within the past 10 years and including neurophysiological evaluations were prioritized.

## 3 Approved indications for the cosmetic use of BoNTA

In the 1970s, when BoNTA was used for the medical treatment of strabismus, it significantly reduced glabellar line wrinkles. This observation became a catalyst for using BoNTA for cosmetic indications (Scott, 1980). The initial cosmetic BoNTA applications involved injections of the upper face in typical sites of manifestations for aging signs in the upper face, including glabellar lines, horizontal forehead lines, and periocular (‘‘crow’s feet’’) wrinkles. Botulinum toxins have eight major serotypes; hitherto, only serotype A has been developed for cosmetic indications. Several formulations are branded and approved for cosmetic indications, including onobotulinum toxin A Onobotulinum toxin A (ONA Botox®/Vistabel®; Allergan Inc., Dublin, Ireland), Abobotulinum toxin A (ABO; Dysport®/Azzalure®/Alluzience®; Ipsen, Paris, France/Galderma, Lausanne, Switzerland), Incobotulinum toxin A (INCO; Xeomin®/Bocouture®, NT 201; Merz Pharmaceuticals GmbH, Frankfurt, Germany) and Prabotulinum toxin A (Jeuveau®, Evolus, Inc., United States) (Phan et al., 2022) Although these formulations have a similar mode of action, they differ in their characteristics, and hence their dosing is not interchangeable (Brin et al., 2014, Frevert, 2015). For this reason, we only refer to typical doses for Onobotulinum toxin A (ONA Botox®/Vistabel®; Allergan Inc., Dublin, Ireland). The number of injection points and dose of BoNTA depend on the needs of each patient. Higher doses result in longer duration and higher efficacy. However, safety from the treatment limits the dose that is used. Therefore, the standard botulinum toxin dosing is usually 20 U for treating the glabellar lines, 24 U for periorbital lines, and 20 U for the frontal muscle.

### 3.1 Glabellar lines

**Fig. 1:**
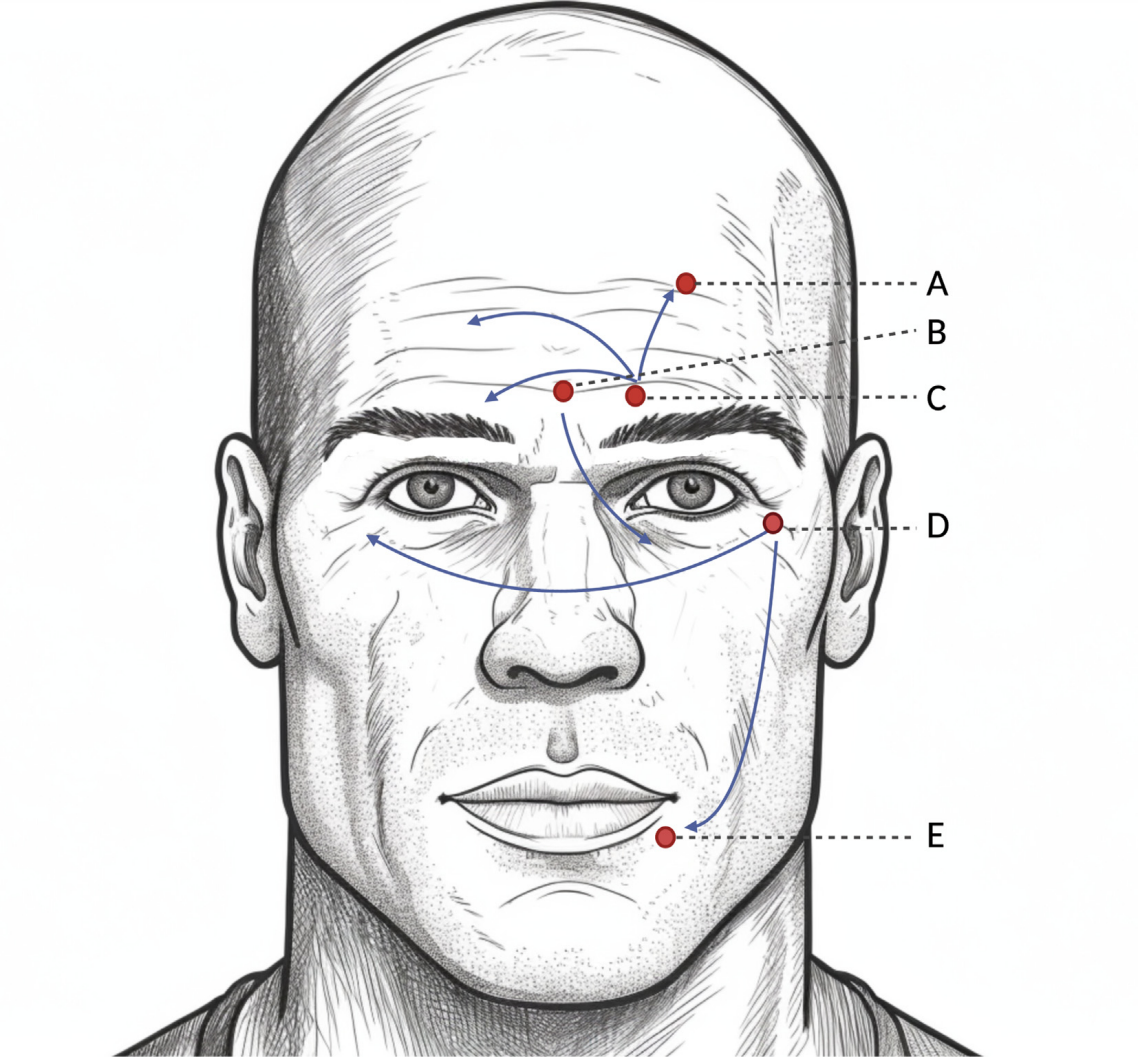
Commonly targeted facial muscles in cosmetic treatment with botulinum toxin. A) Frontal muscle, B) procerus muscle, C) corrugator supercilii muscle, D) orbicularis oculi muscle and E) orbicularis oris muscle. The arrows indicate the neurophysiologically demonstrated spreading of the effect of botulinum toxin to adjacent or contralateral muscles in split face studies (Punga et al., 2015).

Glabellar lines were the first indication for FDA-approved cosmetic use of BoNTA. Four BoNTA preparations are currently approved for improving moderate-to-severe glabellar lines: onabotulinumtoxin A, abobotulinumtoxin A, incobotulinumtoxin A, and prabotulinumtoxin A (3). The muscles contributing to the glabellar muscle complex are the two corrugator superciili muscles and the procerus muscle, which both function as eyebrow depressors (Fig. 1). BoNTA injection into the procerus and corrugator muscles causes muscle relaxation and reduced rhytids between the eyebrows in the glabellar region. In addition to cosmetic improvement, this treatment has been suggested to positively impact the patient's mood and a potential treatment for depression (Wollmer et al., 2022). The most common injection pattern is five injection points of 4 Units resulting in a total of 20 Units.

### 3.2 Crow’s feet (periorbital rhytides)

Other dynamic facial lines are the periorbital rhytides, also known as “crow’s feet”. The successful use of onabotulinumtoxin A has been reported in several studies by reducing periorbital wrinkles (Lowe et al., 2002). The injections should be superficial, respecting the limit of 1 cm from the orbital border and 1.5 cm from the lateral canthus to prevent the product from spreading to undesired areas, reaching, for example, the lateral rectus muscle, innervated by the abducens nerve, which can lead to paresis of this muscle and diplopia (Borba et al., 2022).

### 3.3 Forehead lines (frontalis muscle hyperactivity)

Repeated contractions of the frontalis muscle on the forehead over time cause horizontal wrinkles to form in the overlying skin. When they persist even at rest, they are called hyperfunctioning facial lines. This indication expanded rapidly during the SARS-CoV-2 pandemic due to highlighting the importance of the upper face expression during digital meetings and the focus on the expressions of the upper face with the constant wearing of facial masks (Borba et al., 2022). The frontal facial region can demonstrate expressions without the need for words, expressions such as surprise, anger, joy, and concern. The purpose of BoNTA treatment in the frontalis muscle is to soften expression lines without causing brow ptosis or eliminating the entire upper face expression (Fig. 1).

### 3.4 Off-label indications

BoNTA is being increasingly used off-label for a variety of cosmetic indications. These include treatment of the upper-, mid-, and lower face, such as eyebrow lift, gummy smile, vertical perioral rhytids, and platysmal bands for neck-lift (Jabbour et al., 2017). Most of these indications build on data from case series of cohort studies, and the follow-up of patients is a maximum of 2 weeks post-treatment (Phan et al., 2022). The masseter muscle is the most powerful masticatory muscle, responsible for jaw closing and, thus, chewing (Fig. 1). Due to its more prominent thickness in Asians than in Caucasians, Asian women, in particular, are interested in reducing the masseter muscle volume to improve the lower facial contour (Wu, 2010).

## 4 Short- and long-term neuromuscular effects and side effects of BoNTA

Complications from intramuscular BoNTA injections into the upper face can be divided into localized adverse events and neuromuscular-related adverse events. The most common general adverse events from cosmetic BoNTA injections include pain and bruising, bruising and edema at the injection site, and headache (reviewed in (Sethi et al., 2021)). Neuromuscular-related side effects are less common and include ptosis of the eyelid or brow, usually due to denervation of the levator palpebrae muscle, diplopia, and muscle weakness within and outside the injection area resulting in dysphagia or neck muscle weakness. Serious adverse events, reported hours to weeks after treatment with BoNTA, include anaphylaxis, dysphagia, respiratory insufficiency, and generalized muscle weakness. However, these rare systemic events are only seen at excessively high dosages or in patients with underlying medical conditions.

### 4.1 Short term effects and side effects

Headache is the most common adverse condition reported, followed by injection site reactions such as pain, pruritus, dysesthesia, and bruising. Hypersensitivity reactions and distant spread of the toxin have been reported; however, they are uncommon (Small, 2014). The prevalence of muscular side effects is somewhat different depending on the cosmetic indication for BoNTA injection. Injection for glabellar lines may result in blepharoptosis, eyelid ptosis, and difficulty opening the eyelid. In contrast, injections for frontal rhytidis may result in dysarthria, eyelid ptosis, dysphagia, and difficulty in eye-opening (Sethi et al., 2021).

### 4.2 Expected short term electrophysiological findings in BoNTA treated facial muscles

From mouse studies, it is known that the original nerve terminals cannot undergo measurable exoendocytosis until almost two months after injection of BoNTA, starting already day three post-injection with a dramatic diminution in depolarization-induced exocytosis from the nerve terminals (9). For the glabellar muscles, marked reduction in the corrugator supercili CMAP amplitude correlates well with the dose of onabotulinumtoxin A (Vistabel®) and abotulinumtoxin A (Azzalure®) injected in the same muscle (Alimohammadi et al., 2014, Punga et al., 2016). In female subjects, the baseline amplitude of the corrugator supercilii muscle ranges from 0.8 mV to 1.7 mV (9). After one single standard injection of 20 U onabotulinumtoxin A or 50 U of abotulinumtoxin A in the glabellar area, the CMAP amplitude is approximately reduced to 80% of baseline one day after treatment, to 55% three days after treatment, to 28% of baseline at two weeks, to 35% at one month, to 50% at three months and 60% at six months (Alimohammadi et al., 2014, Punga et al., 2016). Abnormal spontaneous activity on electromyography (EMG) in the glabellar muscles, including positive sharp waves and fibrillations, occur from 2 weeks post-injection and become more pronounced at four weeks. This denervation activity is typically followed by low-amplitude, unstable motor unit potentials or total block of voluntary contraction, i.e., no MUPs were detected by EMG at four weeks post-injection (Alimohammadi et al., 2014). At 12 weeks, denervation activity is typically absent, and instead, signs of ongoing reinnervation are observed, with a maximum EMG interference pattern of approximately 25% activity (Alimohammadi et al., 2014). The EMG pattern is similar in women receiving 20 U and 10 U onabotulinumtoxin A, with neither of the dose groups obtaining maximum contraction capacity at six months, instead being able to activate the muscle with approximately 40% of the initial activity pattern.

### 4.3 Expected electrophysiological findings in surrounding muscles to BoNTA-treated facial muscles

BoNTA can spread to contralateral facial muscles upon unilateral injection of BoNTA in the glabellar or ocular area (Girlanda et al., 1996, Punga et al., 2015) (Fig. 1), which is a critical consideration for evaluating adverse events after BoNT injections, invalidating the interpretation of so-called “split face studies.” Upon unilateral injection of BoNTA in the corrugator supercilii muscle, the CMAP amplitude of the frontal muscle on the same side is reduced by 40% after two weeks and 50% after four weeks, and EMG on the same frontal muscle confirms abnormal spontaneous activity (fibrillations and positive sharp waves), indicating pharmacological denervation in that surrounding muscle (Punga et al., 2015). Further, reduced CMAP amplitude of the contralateral orbicularis oculi or corrugator supercilii muscle is expected upon unilateral BoNTA injection (Girlanda et al., 1996, Punga et al., 2015). Increased neuromuscular jitter measured by concentric needle electrode is expected in the surrounding orbicularis oculi muscle at two weeks following BoNTA injection in the glabellar muscles (Alimohammadi et al., 2014), in the contralateral orbicularis oculi muscle upon unilateral injection in the orbicularis oculi muscle (Girlanda et al., 1996) and in the contralateral frontal muscle upon unilateral injection in the corrugator supercilii muscle (Punga et al., 2015), with higher jitter values upon higher BoNTA doses. The absence of abnormal spontaneous activity on EMG in the orbicularis oculi muscle at two weeks indicates that the subclinical neuromuscular transmission failure does not cause substantial pharmacological denervation as measured by EMG in the typical case of one dose of BoNTA in the glabellar muscles (Alimohammadi et al., 2014). Further, a case illustrated that as little as 4 U of abotulinumtoxin A into the left orbicularis oculi muscle might result in denervation of the left orbicularis oris muscle and the development of left-sided hemifacial paralysis (Punga et al., 2015). In Fig. 1, the spread of BoNTA based on the available scientific literature has been schematized; however, this does not exclude other spreading routes.

### 4.4 Medium term neurophysiological effects and side effects

Skeletal muscles can be divided into “delayed synapsing” or “fast synapsing” muscles based on their response to denervation. BoNT injections into delayed synapsing muscles result in extensive motor nerve sprouting, i.e., reinnervation response, which is absent in fast synapsing muscles (Pun et al., 2002). Facial and bulbar muscles harbor low receptor muscle-specific tyrosine kinase levels and are, therefore, more vulnerable to denervation upon blocked neuromuscular transmission (Punga et al., 2011). Thus, excessive or repeated doses of BoNT may render these muscles atrophic, and unwanted side effects may arise that limit normal facial muscle functions. One main issue regarding cosmetic BoNTA applications is that although widely used, safety studies still need to be improved for people younger than 18 years and older than 65 years of age. Further, the approved cosmetic indications for BoNTA injections are temporary improvement of hyperfunctional facial lines and not repeated or chronic BoNTA use. The efficiency and the safety of repeated injections of BoNTA beyond 12 months have yet to be evaluated; however, despite this, the cosmetic use of BoNTA by far extends this time limit in practice since a 40 or 50% reduction of the CMAP response in the corrugator supercilii muscle lingers on after six months after one single dose of labeled volume or twofold volume of abotulinum toxin A respectively (Punga et al., 2016). Consequently, any new injection in this muscle after as early as three months would severely limit muscle activation and reinnervation.

### 4.5 Long term clinical and physiological effects of BoNTA

BoNTA cleaves a SNAp receptor (SNARE) protein called SNAP-25, located on the inner side of the presynaptic nerve terminal membrane (Blasi et al., 1993). This cleavage, in turn, inhibits the transportation and docking of acetylcholine-carrying vesicles to the presynaptic membrane, inhibiting acetylcholine release into the neuromuscular junction (NMJ) and thus chemical denervation with the consequent inability to perform skeletal muscle contraction. Axonal sprouting and endplate elongation occurs but is believed to be a transient phenomenon not responsible for the termination of the BoNTA effect (de Paiva et al., 1999). These newly formed sprouts, not the initially poisoned NMJs, are the only sites undergoing depolarization-dependent vesicle turnover at the onset of the recovery of nerve-stimulated muscle contraction at day 28 after one injection of BoNTA cervical muscle in mice (de Paiva et al., 1999). Therefore, only new sprouts are responsible for neuromuscular transmission early on, whereas the original terminals cannot undergo measurable exoendocytosis until almost two months after BoNTA injection (de Paiva et al., 1999). However, a second, distinct phase of the rehabilitation process followed with a return of vesicle turnover to the original terminals until up to six months after the BoNTA injection, accompanied by an elimination of the by-then superfluous sprouts (de Paiva et al., 1999). The effects of BT-A injection were long thought to be entirely reversible at the NMJ; however, recent studies on rabbits (Fortuna et al., 2015) and rats (Kaya et al., 2020) indicate long-term changes in skeletal muscle composition. Muscle strength does not recover to baseline values following a six-month recovery period from the last BoNTA injection, most likely due to increased collagen content and reduced active muscle force (Fortuna et al., 2015, Kaya et al., 2020). In a study of two men injected with 20 U onabotulinumtoxin A in the procerus and corrugator supercilii muscles, volumetric muscle analysis revealed up to 48% reduced procerus muscle volume up to 12 months post-injection. In contrast, glabellar line status returned after 6–10 months (Koerte et al., 2013).

### 4.6 Consequences of long-term BoNTA injections for clinical neurophysiology diagnostics

There are several reports in the literature regarding suspected neuromuscular disorder in conjunction with previous cosmetic BoNTA injections, primarily those resembling Myasthenia Gravis (MG) with ptosis and diplopia (Sunness and Kelman, 2004, Farooq et al., 2009, Parikh and Lavin, 2011, Alaraj et al., 2013, Punga and Liik, 2020) or even amyotrophic lateral sclerosis upon injections of repeated BoNTA doses in the bulbar region (Punga and Liik, 2020). Based on the increased use of BoNTA primarily in cosmetic applications, where medical charts may be lacking in hospitals, it is essential to inquire patients about previous BoNTA injections before considering electrophysiological examinations. Otherwise, we risk having a false positive diagnosis of MG with increased jitter on SFEMG in facial muscles, such as the orbicularis oculi or frontal muscles, commonly examined when the referring diagnosis is MG. It is also important to acknowledge that the patient may not know or be willing to reveal all the details of the previous BoNTA treatment. This can result in several unnecessary examinations and tests and a significant psychological burden for the patient. However, systematic electrophysiological investigations of findings in facial muscles after repeated BoNTA injections still need to be improved in the scientific literature. We now know how much reinnervation is possible upon serial injections in the facial muscles.

## 5 Summary

Cosmetic indications for BoNTA injections are increasing globally and are out of reach of most hospitals and clinicians since they are commonly performed in practices by nurses. The resulting disturbed neuromuscular transmission and denervation of facial muscles cause issues for the neurophysiological findings in the diagnostic workup of several neuromuscular disorders. Therefore, we aim to provide a timely review from a neurophysiological perspective to alert neurologists and neurophysiologists to ask all patients coming to this diagnostic workup for previous BoNTA injections. In the future, we need scientific studies evaluating the long-term effects of repeated BoNTA injections in the facial muscles.

## Declaration of Competing Interest

The authors declare the following financial interests/personal relationships which may be considered as potential competing interests: [A.R. Punga and M. Alimohammad have previously received consulting fees from Galderma and M. Alimohammadi is currently receiving consulting fees from Galderma].
